# Metabolic Plasticity in Cancer Cells: Reconnecting Mitochondrial Function to Cancer Control

**DOI:** 10.4172/2157-7013.1000211

**Published:** 2015-06-22

**Authors:** V. Krishnan Ramanujan

**Affiliations:** Metabolic Photonics Laboratory, Department of Surgery, Biomedical Imaging Research Institute, Department of Biomedical Sciences, Samuel Oschin Comprehensive Cancer Institute, Cedars-Sinai Medical Center, 8700 Beverly Blvd., Los Angeles, CA 90048, USA

## Abstract

Anomalous increase in glycolytic activity defines one of the key metabolic alterations in cancer cells. A realization of this feature has led to critical advancements in cancer detection techniques such as positron emission tomography (PET) as well as a number of therapeutic avenues targeting the key glycolytic steps within a cancer cell. A normal healthy cell’s survival relies on a sensitive balance between the primordial glycolysis and a more regulated mitochondrial bioenergetics. The salient difference between these two bioenergetics pathways is that oxygen availability is an obligatory requirement for mitochondrial pathway while glycolysis can function without oxygen. Early observations that some cancer cells up-regulate glycolytic activity even in the presence of oxygen (aerobic glycolysis) led to a hypothesis that such an altered cancer cell metabolism stems from inherent mitochondrial dysfunction. While a general validity of this hypothesis is still being debated, a number of recent research efforts have yielded clarity on the physiological origins of this aerobic glycolysis phenotype in cancer cells. Building on these recent studies, we present a generalized scheme of cancer cell metabolism and propose a novel hypothesis that might rationalize new avenues of cancer intervention.

## The Premise: Riding the Three Waves of Metabolic Switch

**Figure 1:**
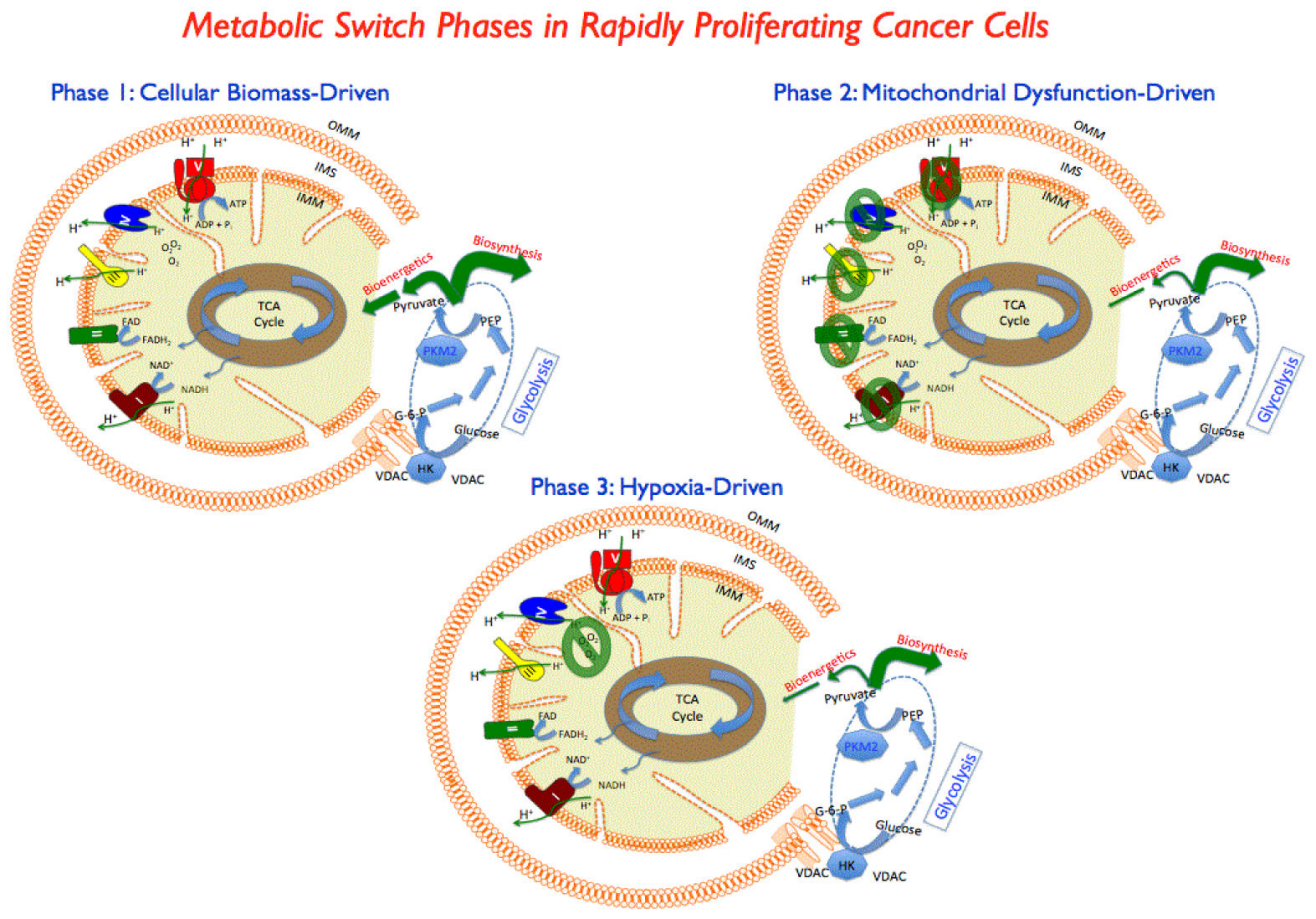
Three distinct phases of metabolic switch in cancer cells: Phase 1 is characterized by driving the glycolytic precursors to aid in an apparent increase in biosynthetic route in response to the need for an increased biomass for the rapidly proliferating transformed cells. Mitochondrial TCA cycle and the electron transport chain, signified by the respiratory complexes I through V, are expected to function without significant loss in function. Phase 2 stems from one or more mitochondrial electron transport complex dysfunction and/or other enzymatic defects – thereby exacerbating glycolytic output. Phase 3 is characterized by oxygen deficit (hypoxia) and mitochondrial respiratory chain output is expected to be minimal despite a functional electron transport chain since the terminal step is deprived of oxygen to impact the bioenergetics. OMM: outer mitochondrial membrane; IMM: inner mitochondrial membrane; IMS: intermembrane space; NADH and FADH2 are the reducing equivalents that feed into the respiratory chain complexes I and II respectively; The rate limiting enzymes hexokinase 2 (HK2) and M2 isoform of pyruvate kinase (PKM2) are shown in the glycolytic pathway. The decision making step for biosynthetic diversion is at the site of PKM2 which in turn, catalyzes the conversion of phosphoenolpyruvate (PEP) to pyruvate. An increased biomass requirement is expected to minimize pyruvate production as compared to normal biosynthetic/ bioenergetic balance.

Transformation of a normal cell to a cancer cell by one or more of the oncogenic events is the first step in the tumorigenesis [1–5]. While the transformation step by itself, can be stochastic, the subsequent survival fate of the cancer cell is determined by three essential factors: (a) an internal rewiring of genetic and metabolic programs within the cancer cell to ensure its *survival* escaping the normal cellular apoptotic programs of eliminating genotoxic stress [3–5]; (b) an alteration in regulatory cell cycle and senescence programs so that the cancer cell not only survives but also continues to proliferate rapidly; and (c) a significant modification of the immediate microenvironment so that the rapidly proliferating tumor biomass is not hindered by a host of regulatory tissue homeostasis programs. In order to survive and proliferate, a cancer cell is therefore obligated to switch from normal metabolic state to a different state that favors its survival - in step with the concomitant cell-autonomous changes as well as the microenvironment changes within the tissue where the cancer cells reside. The first phase of metabolic switch coincides with an apparent increase in glycolytic activity via increased expression of glucose transporters on the cell surface as well as the hexokinases that enable glucose retention by phosphorylation [6–15]. Since ATP is not the limiting factor, this first phase of metabolic switch contributes significantly to increasing the cancer cell biomass by diverting glycolytic precursors to biosynthetic pathways as reviewed elsewhere in detail [16,17]. Notably, this initial phase of metabolic switch does not necessarily require mitochondrial dysfunction and/or oxygen deficit. In fact, this new metabolic demand of increasing cancer cell biomass relies heavily on the substrates such as citrate, alpha ketoglutarate which are the products of mitochondrial TCA cycle enzymatic function [14,18–25]. It has been shown that under glucose limitation, TCA cycle could be reprogrammed to be driven solely by glutamine – thereby generating citrate essential for lipid synthesis [12,26]. It is therefore conceivable that mitochondrial function supports the first phase of metabolic switch in cancer cells at least in biomass accumulation if not in ATP supply. This conjecture has been confirmed in many cancer cells in culture that retain intact mitochondrial function despite an increased glucose metabolism and tumorigenic profile. Mitochondrial dysfunction could potentially arise from a variety of sources including mitochondrial DNA mutations, aberrant TCA cycle and electron transport chain activities, impaired redox balance and anomalous free radical generation/removal rates [14,15,18–19,22–24,27–31]. In cancer cells, these dysfunctional mitochondria could further exacerbate the glycolytic flux to sustain survival/proliferation demands thereby addicting them to glucose and glutamine metabolism pathways. This constitutes the second phase of metabolic switch in cancer cells, characterized by mitochondrial dysfunction. It is possible that the mitochondrial dysfunction in the second metabolic switch phase could primarily stem from compromised electron transport chain activities (oxidative phosphorylation arm) rather than the TCA cycle activities. As the cancer cell biomass increases in size and shape – exceeding that can be supported by normal vascular development program and nutrient supply, then oxygen availability becomes a critical issue within the solid tumors [10,11,32]. This leads to hypoxia and subsequently, the third phase of metabolic switch in cancer cells rendering them to up-regulate glycolytic genes in response to hypoxia. As we can see, this metabolic switch phase is distinctly different from the other two phases in the fact that reduced oxygen tension is its physiological origin. In summary, metabolic switch within a cancer cell could occur in three distinct phases: the first phase characterized by biomass-driven aerobic glycolysis, the second phase characterized by mitochondrial-dysfunction-driven aerobic glycolysis and finally the third phase characterized by hypoxia-driven anaerobic glycolysis. It can be speculated that every proliferating cancer cell, in its lifetime, has an opportunity to ride one or more of these three waves of metabolic switch while contributing to the overall tumor metabolism. There is an immediate practical utility of the classification of cancer cell metabolic switch phases as summarized here (Figure 1). First of all, it helps rationalizing the discrepancies in experimental observations. As can be reasoned here, Warburg’s original hypothesis of the link between aerobic glycolysis and mitochondrial dysfunction pertains only to the second phase of metabolic switch and thus is only a special case of this general scenario of metabolic switch [16,24,27,33]. This realization helps explain why some cancer cells do not display mitochondrial dysfunctions despite manifesting hyperglycolytic and/or tumorigenic profiles [34–36]. These cancer cells can now be understood as displaying only the first phase of metabolic switch thereby retaining mitochondrial function in order to support their tumorigenic needs. Genetic evidence for this conjecture comes from studies in c-myc driven tumors where mitochondrial function is in fact increased so as to support biomass accumulation as described above [1,12,37–39]. Thus any meaningful discussion of cancer cell adaptation may need to incorporate the different metabolic switch phases described here. Spatial and temporal distribution of oxygen tension can vary significantly across the solid tumors thereby leading to significant heterogeneity in the metabolic switch profiles within the tumor mass. Since oxygen availability has been shown to influence carbon metabolic fluxes, bioenergetics profiles as well as transcriptional profiles, one could further speculate that the tumor heterogeneity commonly observed might be intricately tied to the metabolic heterogeneity arising from cancer cells displaying differential metabolic switch phenotype. Experimental evidence for this conjecture can be sought in a study that investigated the spatial distribution of cancer cells in relation to their distance from the blood flow and how lactate generated by hypoxic cells distal to the blood vessel could potentially feed, via conversion to pyruvate, into mitochondrial respiration in aerobic cells closer to the blood vessel [40]. We therefore have an evolving picture of solid tumors as dynamic entities of cancer biomass with mixed continually changing metabolic switch characteristics. Pertinent to the clinical challenge of tumor control, it will be intriguing to investigate if the different metabolic switch phases can be predictably mitigated/reversed and if these metabolic switch phases could be exploited for their unique vulnerabilities so as to achieve therapeutic benefits.

## The Metabolic Plasticity Hypothesis

The three phases of metabolic switch phenotype described above depend on a multitude of factors including the nature of oncogenic drive (e.g., c-myc or MET or PI3K mutations), organ site (breast or liver or brain) and the overall physiological status (e.g., diabetes, obesity etc.,) of the individual [41,42] Warburg’s original idea that aerobic glycolysis in cancer cells stems from irreversible mitochondrial respiration injury has not been found to be applicable in all the cancers. As rationalized above, the aerobic glycolysis regime described in Warburg’s theory can be understood only as a special case of the generalized metabolic switch description. Recent studies of metabolic switch phenotype in human cells mediated by mitochondrial complex I dysfunction - point out that in situations of moderate mitochondrial dysfunction, it is indeed possible to reverse metabolic switch phenotype via modulating the redox poise in these cells [23,43]. In another study, metabolic adaptation to long-term mitochondrial modulation in aggressive breast cancer cells was found to mitigate tumor growth in vitro and in preclinical animal models via mitigating aerobic glycolysis and via improving mitochondrial function [44]. These observations pose an interesting possibility that at least some cancer cells may display a “metabolic plasticity” regime where the aerobic glycolysis and/or mitochondrial characteristics could either be reversed or reprogrammed. Common to all human cancers is the propensity of individual cancer cells to adapt to both intrinsic, cell-autonomous metabolic needs as well as the global physiological demands –which together increase their survival. Studies show that cancer cells display a wide variety of adaptation strategies, contributing to resistance mechanisms that they manifest in response to therapeutic modalities. Partial failures in the traditional “cell killing” approaches such as chemotherapy and radiation therapy point out to a need for alternative strategies. Towards this direction, targeting the cancer cell adaptation to the metabolic switch phases offers a viable avenue where it is possible to “prime” the cancer cells by mitochondrial normalization so that normal regulatory processes (such as cell cycle, apoptosis and growth factor signaling) can help mitigate primary cancer cell growth and help improving therapeutic efficacy by mitochondrial apoptosis.

## Implications of the Metabolic Plasticity Hypothesis for Cancer Detection and Treatment

**Figure 2:**
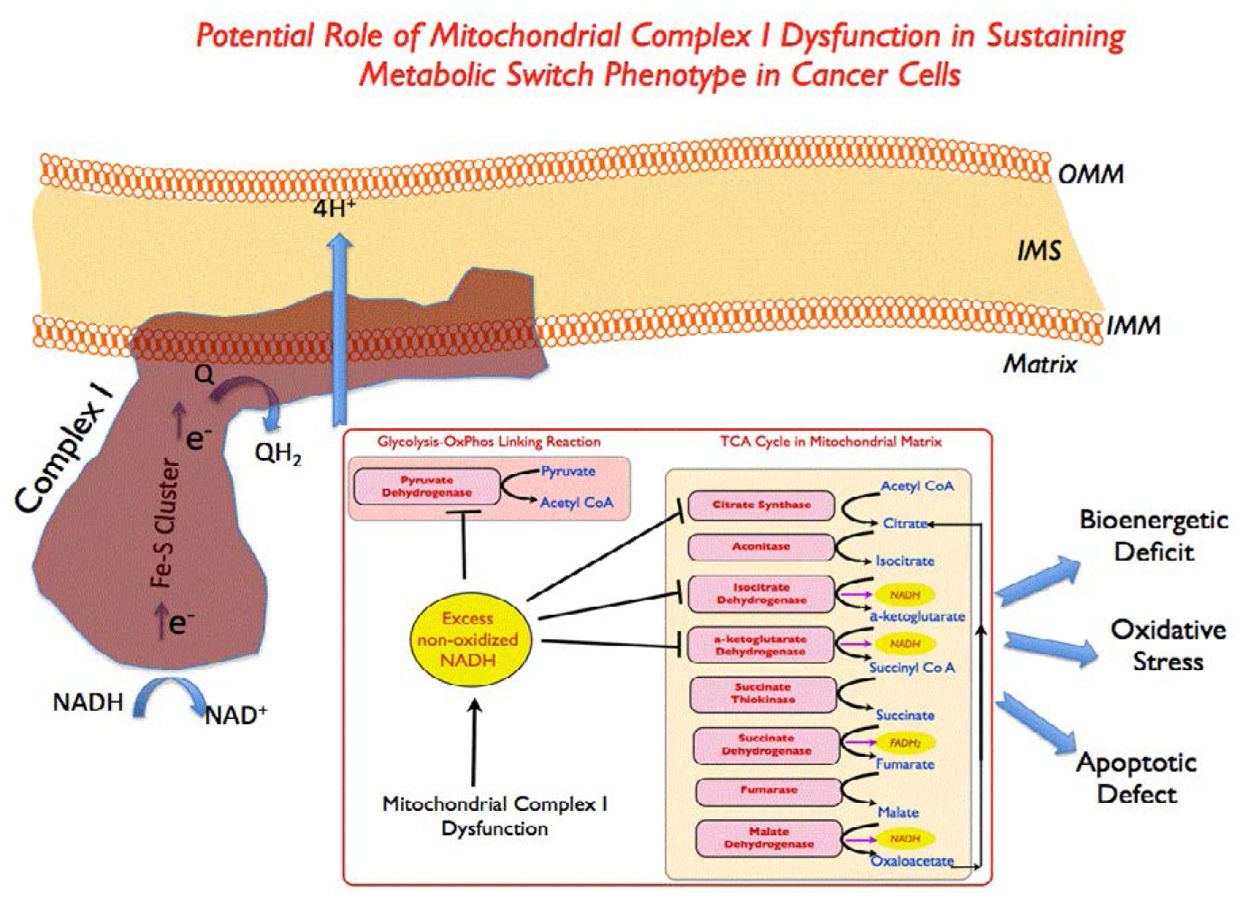
Putative schematic of mitochondrial complex I function in the decision making of cancer cell metabolic switch phenotype: Mitochondrial complex I serves as a gate keeper between the glycolytic/TCA cycle network and the electron transport chain activity in the mitochondria. This is the largest enzyme (45 subunits in mammalian enzyme) of the electron transport chain. Electrons generated via oxidation of NADH to NAD+ are transported via the iron-sulfur clusters within the peripheral arm of mitochondrial complex I (as shown) to reduce ubiquinol (Q to QH2) thereby transporting 4 protons from the matrix to the intermembrane space thereby significantly contributing to the mitochondrial membrane potential that is eventually used for ATP synthesis. Any dysfunction arising from structure assembly and/or functional aberration in the Complex I is expected to build up excess NADH at the first step of enzyme activity which will in turn, lead to a significant imbalance in the associated metabolic pathways as shown here. Overall mitochondrial dysfunction can lead to bioenergetics deficit, oxidative stress due to an increased free radical generation and apoptotic defects – all contributing directly to sustain the metabolic switch phenotype in cancer cells.

Metabolic plasticity as described above, can be understood as the amenability of the cancer cells to partially or completely reverse their metabolic switch phenotype. Since aerobic glycolysis is intricately tied to biomass accumulation, targeting metabolic plasticity regime may therefore provide a unique opportunity to normalize the aberrant growth potential in cancer cells [1,2,4,6,10,25,28,31,42,45–49] This situation is fundamentally different from the well known cancer cell adaptation pathways (hypoxia adaptation, chemoresistance etc.,) that inherently confer survival and proliferative advantages to the cancer cells. Most of the efforts in the past for targeting metabolic switch phenotype have been primarily focused on glycolytic targets or on oncogenic pathways (such as PI3K-AKT axis, mTOR) that upregulate glycolytic pathway. While these drug targets had shown promise in preclinical setting or Phase 1 clinical trials, their success in Phase 2/3 clinical trials have been often limited [40,50]. In recent times, the above situation has led many researchers to focus on the mitochondrial machinery for understanding cancer cell metabolism as well as for potential drug targets. This includes studies on mitochondrial TCA cycle enzymes such as alphaketoglutarate, isocitrate dehydrogenase as well as a few modulators of electron transport chain components such as metformin. In this context, our laboratory has been exploring the fundamental role of mitochondrial complex I, the largest enzyme complex of the human mitochondrial electron transport chain [51–54]. Mitochondrial complex I functions in regulating redox/ROS status in mammalian cells. Excess, non-oxidized NADH can lead to an imbalance in Glycolysis - TCA Cycle-Electron transport chain (GTE) network. By virtue of being the first step in ETC, mitochondrial complex I has the potential to be the gate-keeper for the cell-fate decision between glycolysis and OxPhos [51]. Since individual ETC complexes are mutually linked and tightly regulated, mitochondrial complex I dysfunction can lead to an overall compromised mitochondrial function in the cells. These mitochondrial defects could potentially exacerbate the second phase of metabolic switch phenotype as described earlier. Identifying putative mitochondrial complex I defects in cancer cells and devising strategies to improve overall mitochondrial function in cancer cells can be an attractive opportunity for influencing the metabolic switch phenotype in cancer cells. Preliminary results supporting this notion came from our recent studies where we observed that human, triplenegative breast cancer cells displayed different degrees of mitochondrial complex I function and those cells which showed a better amenability for complex I modulation also displayed a greater degree of metabolic plasticity (i.e., increased mitochondrial function and decreased aerobic glycolysis phenotype) [44] (Figure 2). Furthermore the aggressive breast cancer cells overexpressing a critical catalytic subunit (NDUFS3) of mitochondrial complex I also showed a clear trend for mitochondrial normalization and a concomitant growth reduction. More recently, mitochondrial complex I function, via the (NADH/NAD+) ratio, has been shown to be a determining factor in breast cancer growth and progression [23]. Together these studies reveal an unexplored, critical role of mitochondrial complex I in cancer cell metabolism and plausibly, cancer control. It is indeed possible that other electron transport chain enzymes could play an equally important role in cancer growth as emerging from other recent studies in the field. Before this idea of metabolic plasticity or reversibility of metabolic switch phenotype can be put to practical use, there is an acute need to answer a few critical questions: what are the distinct biomarkers indicative of metabolic plasticity regime in cancer cells ? Are all cancers amenable to this strategy of targeting metabolic plasticity for growth reduction ? Is the reversal in metabolic switch phenotype predictable and controllable ? In the absence of any large scale studies addressing these questions, it will be informative to carry out systematic studies for identifying global proteomic and metabolomic signatures of metabolic plasticity in cancer cells. It is highly likely that these signatures will vary depending on the organ type and the oncogenic drive in different cancer subtypes. However, identifying the differential proteomic signatures as well as common overlapping schemes in different cancer models will inform us as the general validity of metabolic plasticity hypothesis as well as some practical guideline for screening the cancers for their potential amenability for metabolic intervention. From the solid tumor point of view, targeting the aforementioned metabolic plasticity regime in the cancer cells will be largely influenced by the stromal components as well as immune/angiogenic factors. In step with recent realization that recurrent cancers have distinct metabolic profiles that are different from the primary cancers, one could ask if loss of metabolic plasticity regime could serve as a biological reason for the increased aggressiveness of the recurrent cancers.

In conclusion, advancements over the last two decades in cancer diagnostics and therapeutic targeting have profoundly revolutionized our ability to understand and intervene human cancers. Chemoresistance and recurrent cancers after surgery – are the main bottlenecks in cancer management. Towards this direction, priming the cancer cells by strategic targeting of metabolic plasticity regime via mitochondrial normalization – could be an alternative approach for decreasing primary tumor growth and improving therapeutic efficacy.
